# Conventional, organic, and regenerative organic farming differentially shape nutritional and phytochemical profile in beetroot (*Beta vulgaris*) and its commercial powder products

**DOI:** 10.3389/fnut.2026.1874417

**Published:** 2026-08-10

**Authors:** Surbhi Verma, Ying Xi, Muhammad Ahsin, Tiffany Evans, Stephan van Vliet

**Affiliations:** 1 Plants, Soils, and Climate Science, Utah State University, Logan, UT, United States; 2 Center for Human Nutrition Studies, Utah State University, Logan, UT, United States; 3 Utah State University Analytical Laboratory, Utah State University, Logan, UT, United States

**Keywords:** beetroot supplements, betalains, farming practices, functional foods, nitrates, root vegetables, ultra-high performance liquid chromatography-mass spectroscopy

## Abstract

Beetroots are increasingly recognized as a functional food; however, the extent to which different farming practices influence their primary nutrients, including minerals, sugars, and protein, and secondary metabolites, including phenolics, betalains, and nitrates, remains poorly understood. To address this gap, we analyzed fresh beetroot (*Beta vulgaris*) samples collected from a conventional, organic, and Regenerative Organic Certified (ROC^™^) farm (*n* = 12 per farming system) in Southwest Oregon, USA, along with beetroot powder from five commercially available brands samples (*n* = 6 samples per brand). Fresh organic and ROC^™^ beetroots contained approximately 53% higher levels of non-flavonoid polyphenols compared with conventional beetroots (all, *p* < 0.01) but did not differ significantly from each other (*p* = 0.99). Total betalains were approximately 105 and 61% higher in ROC^™^ and conventional fresh beetroots compared with organic beetroots (all, *p* ≤ 0.01), respectively, although ROC^™^ and conventional samples did not differ from each other (*p* = 0.08). Nitrate concentrations in organic beetroots were approximately 20% higher than ROC^™^ and 44% higher than conventional beetroots (both, *p* ≤ 0.03). Mineral concentrations varied across groups without clear directionality; sugars were higher in organic and ROC^™^ beetroots, whereas protein was lower in ROC^™^ beetroots (all, *p* < 0.001). Among commercial beetroot powders, organic powders had the lowest polyphenol concentrations (*p* < 0.001), sugar concentrations were lowest in the conventional powder (all, *p* < 0.01), while nitrate, betalain, mineral, and macronutrient concentrations showed no clear trend. These findings suggest that organic and ROC^™^ production systems in fresh beetroots are associated with higher levels of bioactive phytonutrients, while also highlighting that nutrient composition in commercial powdered beetroot products is influenced by sourcing factors, local agroecological conditions, and post-harvest processing.

## 1 Introduction

Root vegetables are an important part of global diets, constituting a rich source of vitamins, minerals, and phytonutrients such as polyphenols, flavonoids, and other bioactive compounds with ascribed antioxidant, anti-microbial, anti-diabetic, and anti-cancerous effects (1). Additionally, beetroot powders and juices are often marketed as health- and performance promoting supplements due to their nitrate content (2). Beetroots (*Beta vulgaris*) are classified botanically as an herbaceous biennial of the *Chenopodiaceae* family and are considered primarily a cool-season vegetable crop. The beetroot varieties still cultivated today appeared in the late 16th century and are believed to have originated from a historic North African root vegetable (*Beta vulgaris subsp. maritima*) (3). It is broadly accepted that climate, soil types, cultivars, fertilizers, irrigation practices, the use of pesticides and herbicides, maturity at harvest, and postharvest practices can impact the nutritional composition of beetroots (4); however, data on bioactive compounds (phenolics, betalains, nitrates etc.) in beetroots and their functional products (e.g., beetroot powders) grown under different farming practices is still limited (5), especially in regard to regenerative organic (ROC) practices.

There is currently a growing interest in regenerative agriculture to reduce the environmental externalities of farming while sustaining productivity and improving the nutritional value of foods (6). Although many conventional farming systems are efficient and productive in the near term, there is growing concern that their reliance on synthetic inputs may contribute to long-term degradation of soil health and ecosystem function (7). The considerable reliance on synthetic fertilizer and pesticide inputs, along with monoculture cropping, can eventually lead to greater dependency on inputs, concomitantly initiating a potential cycle of diminishing returns over time in cropping systems (8).

In parallel, global population growth and dietary demands have placed additional pressure on agricultural systems, further amplifying the need for maintaining productivity while improving the nutrient density of foods (8) as two billion people worldwide are affected by micronutrient deficiencies (9). This underscores the growing interest in adopting ecologically conscious growing practices including organic, and more recently, regenerative organic agriculture (10).

While contemporary organic agriculture focuses on eliminating synthetic pesticides and fertilizers to reduce environmental and health risks, it does not necessarily require practices that actively restore ecosystem function and soil health, although it is argued that organic agriculture was conceived with those goals in mind (11), and various organic farmers still abide by those goals today. Nonetheless, regenerative organic agriculture extends organic standards by emphasizing soil health, biodiversity, carbon sequestration, ecosystem resilience, and social fairness through outcome-based practices and metrics (12). Regenerative Organic Certified^®^ (ROC^™^) builds on USDA organic certification by requiring additional practices aimed at restoring soil health and the broader farm ecosystem, and may include practices such as cover cropping, crop rotation, reduced or no tillage, compost application, and avoidance of persistent chemical pesticides and petroleum-based fertilizers.

A growing number of publications have highlighted the potential of improving the nutrient density of crops by practicing regenerative agricultural practices (6, 13, 14). In particular, there is initial evidence that crops grown on regenerative farms exhibit greater concentrations of certain minerals and total phenolics than crops from conventional farms, which corresponded with improvements in organic carbon soil on the regenerative farms (7). Nonetheless, inconsistent results are observed when comparing nutritional profiles of crops from organic and conventional farming practices as reviewed by Dangour (15) and Herencia (16); however, most studies have focused on primary nutrients (protein, vitamins, minerals) as opposed to secondary phytonutrient compounds (e.g., phenolics). As primary nutrients (e.g., nitrogen and mineral fertilizers) are frequently used in conventional systems, limited differences may be expected in protein and minerals. Importantly the extent to which regenerative agricultural practices influence crop nutritional quality across broader classes of nutrients has not been fully explored.

Therefore, the objective of this study was to compare primary (protein, sugar, and minerals) and secondary (polyphenols, betalains, and nitrates) nutritional composition of (1) fresh beetroots cultivated in silty loam soils in Southwest Oregon, USA; and (2) powdered beetroot products sourced from five different brands available in the commercial market place, with all fresh and powdered beetroot products originating from three agricultural production systems: conventional, organic, and ROC^™^. To our knowledge, this study is the first to extend conventional-versus-organic comparisons of beetroot by including a Regenerative Organic Certified (ROC^™^) production system, and by evaluating both fresh beetroots and commercially available beetroot powders. In doing so, this study addresses an important gap in understanding how production system and commercial sourcing may influence the nutritional and phytochemical profiles of beetroot and beetroot products available to consumers.

## 2 Methods

### 2.1 Beetroot sample material sources

Fresh beetroots were collected from growers producing organic and ROC^™^ beetroots in 2024 and from a local farmers market selling conventional beetroots in 2025, all within the Grants Pass region of Southwest Oregon, USA. For the ROC^™^ beetroots, seeds were sown directly with 28-inch row spacing and 4-inch in-row spacing under full sun conditions in July 2024 while harvested in December 2024. Prior to planting, on-farm compost was applied at a rate of 10 yards per acre, approximately 3 weeks before seeding. No additional organic fertilizer was applied during the growing season. The field had previously undergone a 3-year rotation with perennial cover crops, primarily oats and red clover, to enhance soil organic matter and nitrogen availability via leguminous fixation. Weed management was conducted using a two-pass tractor row crop cultivator to control inter-row weed growth, followed by one round of intra-row hand weeding with a garden hoe. Beets were harvested in the early morning using a commercial potato harvester and cleaned post-harvest in a barrel tumbler with clean well water. Organic beetroots were sourced from a local grower in the same region. Based on the available information from the grower, the organic farm was cultivated using cover cropping and compost applications like in the ROC^™^ system; however, a key difference is that organic fields received granular/pelletized organic fertilizer either before planting or during the active growing period—an input not used in the ROC^™^ system described above. The conventional beetroots were grown using nitrogen, phosphorus, and potassium (NPK)-based fertilizer and included applications of herbicides, fungicides, and insecticides. These were purchased from the same local farmers’ market in Grants Pass, Oregon; however, further detailed information on seed source and field management was not available.

Upon harvest/collection, randomly selected individual fresh beetroots (*n* = 12 collected per production system/source) were sent to the Center for Human Nutrition Studies, where samples were pulverized under liquid nitrogen and lyophilized. All 12 beetroot samples per production system were treated as individual samples. Additionally, commercial beetroot powders were sourced from five commercial brands, including one ROC^™^, three organic, and one conventional product. Selection was based on market availability and label-indicated production system as conventional, organic, or ROC^™^. The conventional beetroot powder originated from Mexico; organic powder 1 originated from China; organic powder 2 had an unknown country of origin; organic powder 3 originated from the United States; and the ROC^™^ powder originated from the United States, from the same Oregon farm as the fresh ROC^™^ beetroots. Processing information was available from product labels or supplier-provided information. Organic powder 1 was reported as spray-dried, whereas organic powder 3 was dried using fast-drying infrared technology. The ROC^™^ beetroot powder was dried at 100 °F, while drying information was not available for the conventional powder or organic powder 2. For each of the five commercial products, six individual units were obtained (*n* = 6 bags or bottles per brand), resulting in a total of 30 commercial beetroot powder samples. All samples were stored at −80 °C prior to analysis.

### 2.2 Phenolic and betalains analysis

Ultra-high Performance Liquid Chromatography-Mass Spectroscopy (UHPLC–MS) grade acetonitrile (ACN), methanol (MeOH), tert-Butyl methyl ether (MTBE), formic acid (FA), and water (H_2_O) (all, Supelco LiChrosolv^®^) were ordered from Sigma-Aldrich (St. Louis, MO, USA) for extractions. Non-labeled standards of compounds and stable-isotopically labeled (IS) standards including 4-hydroxybenzoic acid-^13^C_6_, apigenin-D_5_, benzoic acid-D_5_, genistein-D_5_, phenol-^13^C_6_, and resveratrol-^13^C_6_ were purchased from Sigma-Aldrich (St. Louis, MO, USA) and/or Cayman Chemical (Ann Arbor, MI). All individual compounds were dissolved at a stock concentration of 1 to 10 mM in the reagents mentioned (or a combination thereof) depending on their solubility. All compounds were optimized by direct infusion after having been diluted to 1 μM to determine optimal MS-parameters, such as declustering potential (Q0D), collision energy (CE), cell exit potential (CXP), and for two Multiple Reaction Monitoring (MRM) transitions (quantifier/qualifier).

Subsequently, 30 mg of lyophilized fresh beetroot samples (*n* = 12 per farming system) and powdered beetroots (*n* = 6 per product) were weighed for extraction. The extraction was conducted using 1,250 μL of internal standard spiked buffer MTBE:MeOH (2:1, v:v), followed by sample lysis at 30 oscillations/s (Qiagen Retsch Tissue Lyser II) with a 5 mm stainless-steel bead, and sonication in an ice bath for 20 min. Extracts were then stored at −80 °C overnight and MTBE was discarded the next day after inducing phase separation using 750 μL of water. Aqueous phases were centrifuged at 16,000 rcf and 4 °C for 10 min, filtered through 0.22 μm PTFE filters, and stored in amber HPLC glass vials at −80 °C until the time of injection. Protein pellets were washed with MeOH, hydrolyzed in 2 M NaOH and incubated for 16 h at 30 °C, followed by pH adjustment to 3 with formic acid, sonication at room temperature, centrifugation at 16,000 rcf (4 °C) for 10 min, and filtration through PTFE (polytetrafluoroethylene) filters. Bound samples then underwent solid-phase extraction (SPE) using Strata-X-Pro (Phenomenex, Torrance, CA) as per manufacturer protocol, and were stored in amber HPLC glass vials at −80 °C until injection. A SCIEX Hybrid Triple Quad^™^ 7500 System-QTRAP^®^ (QQQ-MS) (Framingham, MA) coupled to a Shimadzu Nexera 40 Series LC system (Kyoto, Japan) (UHPLC) was used for MRM with conditions described by Pereira et al. (17).

### 2.3 Macronutrients and mineral analysis

Mineral content was determined using a modified nitric/hydrogen peroxide wet ashing method at Utah State University Analytical Laboratories (18). Five hundred mg per sample was weighed in 20 mL digestion tubes with 6.0 mL concentrated nitric acid (Fisher Certified ACS Plus). After 10 min at room temperature, samples were heated to 80 °C for 10 min, followed by 2.0 mL of 30% H₂O₂ (Fisher ACS Grade). Digestion continued at 130 °C until volume reduced to ~2.0–3.0 mL. Elemental detection and quantification were performed via inductively coupled plasma–atomic emission spectrometry (iCAP 6300 ICP-AES; Thermo Electron, Waltham, MA, USA), operated in radial mode using a cyclonic spray chamber with a nebulizer flow rate of 0.69 L/min. Total protein content (N × 6.25) was determined via nitrogen analysis using the Dumas combustion method by detection of nitrogen gas as described (19). Total soluble sugar was evaluated using Brix according to AOAC 932.13 (20).

### 2.4 Nitrates analysis

Total nitrate content in the samples was quantified using a Nitrate/Nitrite Colorimetric Assay Kit (Cayman Chemical, Ann Arbor, MI) using a two-step enzymatic reduction protocol. Eighty μL of each standard and PBS (phosphate-buffered saline) extracted sample (20 mg of sample) was pipetted into a 96-well microplate, followed by the addition of 10 μL each of reconstituted nitrate reductase enzyme and cofactors. The plate was subsequently incubated at room temperature for 3 h, after which 50 μL each of Griess Reagent I and II were added sequentially. Absorbance was measured at 540 nm using a microplate reader. A standard curve prepared using known nitrate concentrations (0.36–2.5 μg/mL) was used to calculate nitrate levels in the samples. All measurements were performed duplicate and values were averaged.

### 2.5 Statistical analysis

Statistical analysis was performed using GraphPad Prism (v.9.0) to obtain mean ± standard error of mean (SEM) values. One-way analysis of variance (ANOVA) followed by Tukey’s *post hoc* test was conducted to determine statistically significant differences (*p* < 0.05) in individual nutrient profiles among fresh beetroot (*n* = 12/group) and commercial beetroot powder (*n* = 6/group) samples. Phytochemical/antioxidant data was further analyzed using MetaboAnalyst 6.0^1^ as described previously (21). Supervised partial least squares discriminant analysis (PLS-DA) was performed and the variable importance in projection (VIP) score from component 1 was used to rank metabolite contributions to group separation. The top 30 metabolites with the highest VIP scores were selected as key discriminant features for further analysis. A hierarchical clustering heatmap was created using Euclidean distance as the distance metric, and Ward’s method as a clustering method based on PLS-DA VIP to visualize top 30 metabolites that contributed to differences between groups.

## 3 Results and discussion

### 3.1 Fresh beetroots

**Figure 1:**
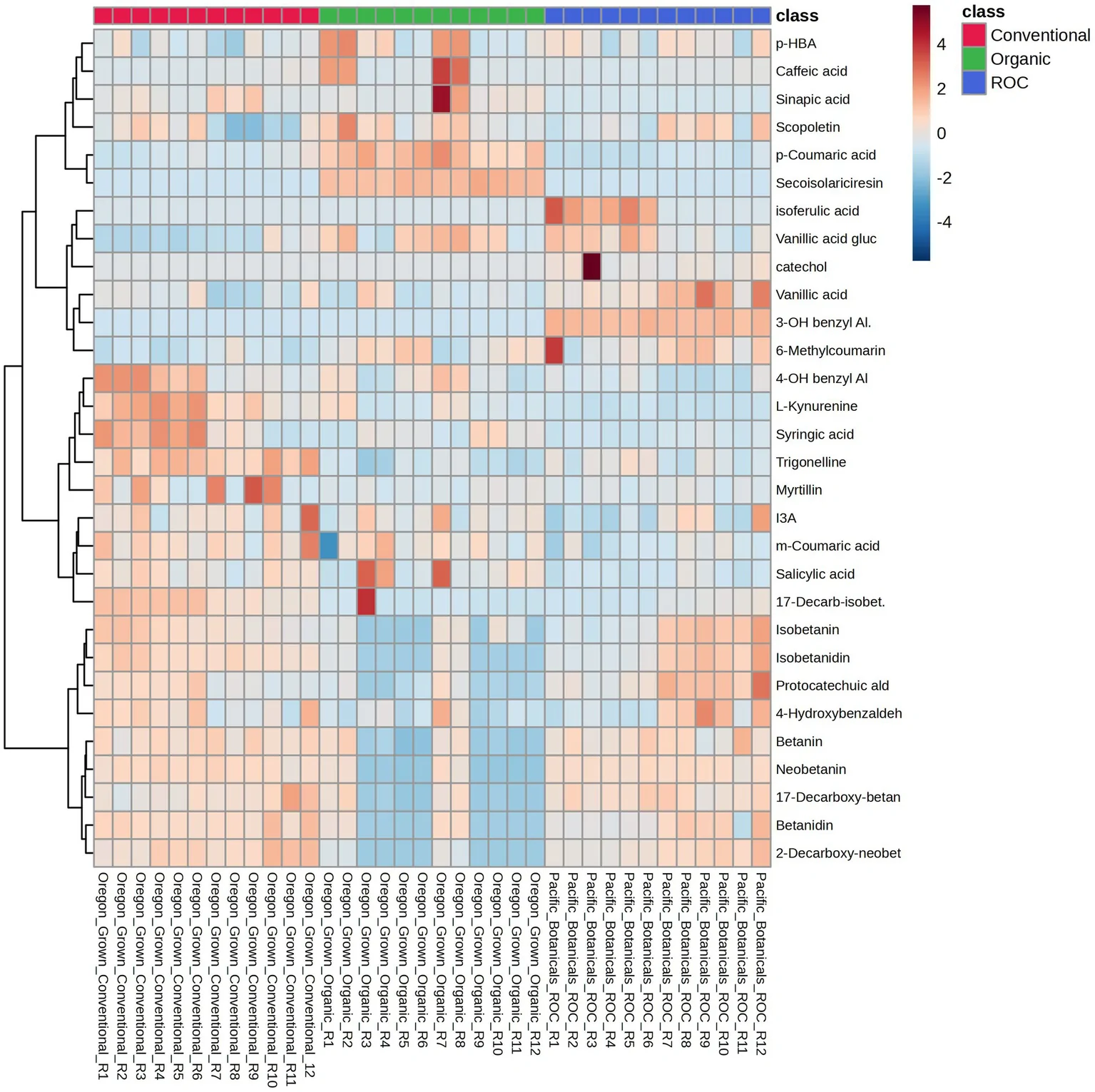
Heat maps illustrate the hierarchical clustering of top 30 plant secondary metabolites driving the group separation (polyphenols and betalains), among beetroot grown under different farming systems. The heat map was created using Euclidean distance as the distance metric, and Ward’s method as a clustering method based on partial least squares-discriminant analysis, variable importance in projection (PLS-DA VIP) score. Red (intensity ranges from 0 to 1) means higher abundance of the corresponding metabolite, whereas blue means lower abundance (intensity ranges from −1 to 0). The numbers below the heatmap represent grouped samples (*n* = 12 for each group).

**Figure 2:**
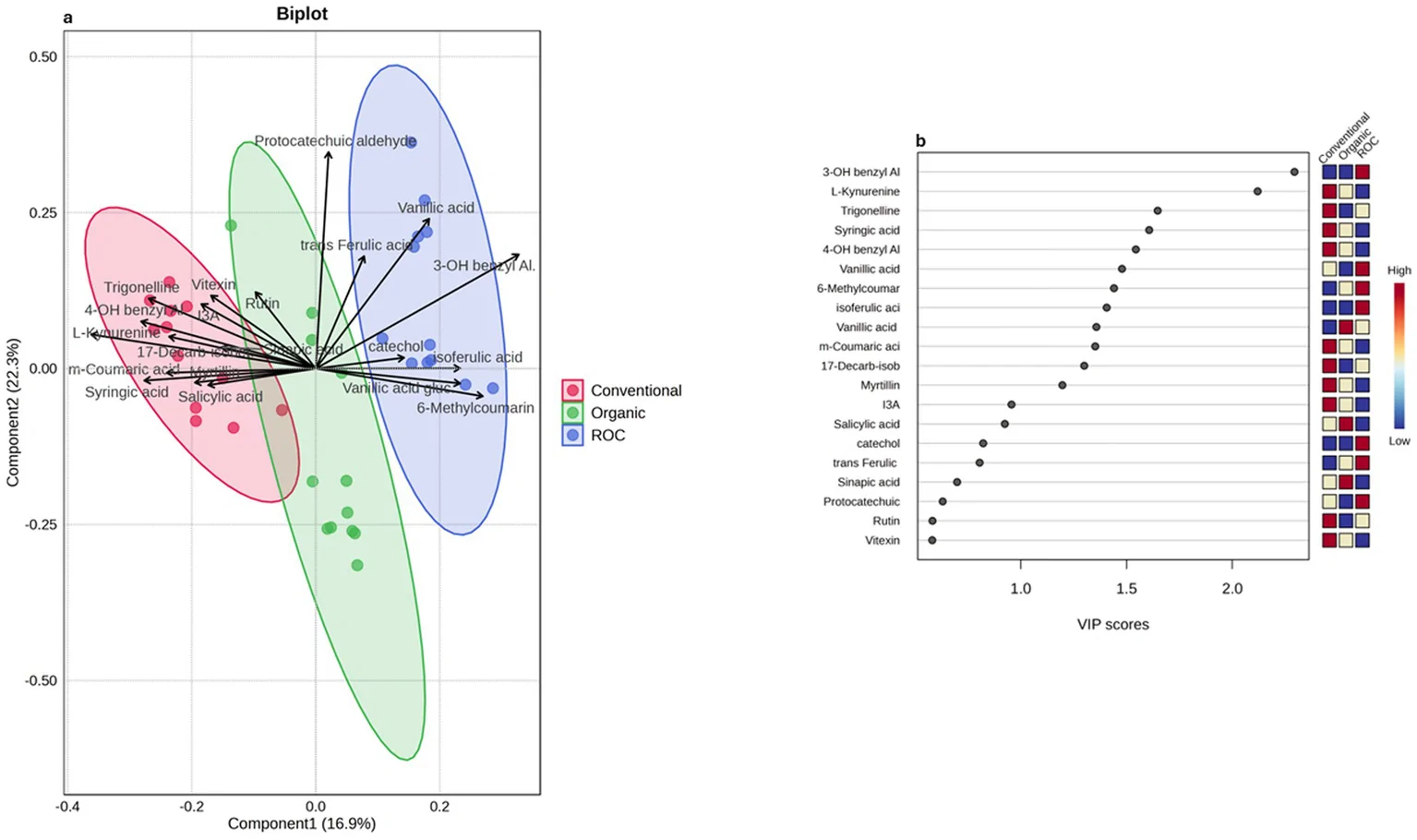
Partial Least Squares Discriminant Analysis (PLS-DA) biplot **(a)** Based on the mean concentrations and **(b)** Variable Importance in Projection (VIP) score plot highlighting top 20 plant secondary metabolites driving the group separation, detected using UHPLC–MS/MS technique. The beetroot replications (colored dots) clustered together share similar metabolic profiles and species which are farther apart have distinct metabolic profiles.

A total of 29 phenolics and related compounds, and eight betalains were quantified in fresh beetroot samples. Figure 1 highlights the top 30 plant secondary metabolites (phenols and betalains) differentiating the three groups of beetroots (conventional, organic, and ROC^™^). Partial Least Squares Discriminant Analysis (PLS-DA) analysis of secondary nutrients elucidated approximately 39.2% of total variance between samples, with component 1 explaining 16.9% variability and component 2 representing 22.3% variability, highlighting a distinct phytonutrient profile among the three production systems (Figure 2a). The direction and length of the vectors in the biplot indicate the contribution of each metabolite towards separation amongst the three production systems. Clustering of conventional beetroots is associated with trigonelline, 4-hydroxybenzyl alcohol, L-kynurenine, *p*-coumaric acid, and syringic acid, while clustering of regenerative organic samples indicated association with vanillic acid, 3-hydroxybenzyl alcohol, isoferulic acid, and 6-methylcoumarin. In contrast, organic beetroot samples showed partially overlapping profiles with both conventional and ROC^™^ beetroots. The VIP plot indicates the metabolites that contribute to the differentiation among beetroot samples, and higher VIP scores indicate the metabolites playing the most significant roles in discriminating between different groups of beetroots (Figure 2b).

#### 3.1.1 Betalains

Betalains are water-soluble nitrogen-containing pigments derived from the amino acid tyrosine. Betalains are found in plants belonging to the *Caryophyllales* genus (which includes beetroots) in which they take the place of anthocyanin pigments (22). There are two main types of betalains, namely betacyanins (red violet) and betaxanthins (yellow orange). Beetroots are characterized by betacyanins such as betanin, isobetanin, neobetain, and 17-decarboxy-betanin. Betacyanins have been studied for various health-promoting properties, including being antioxidative, anti-inflammatory, anti-cancer, blood pressure and lipid-lowering, and hepatoprotective (23).

**Figure 3:**
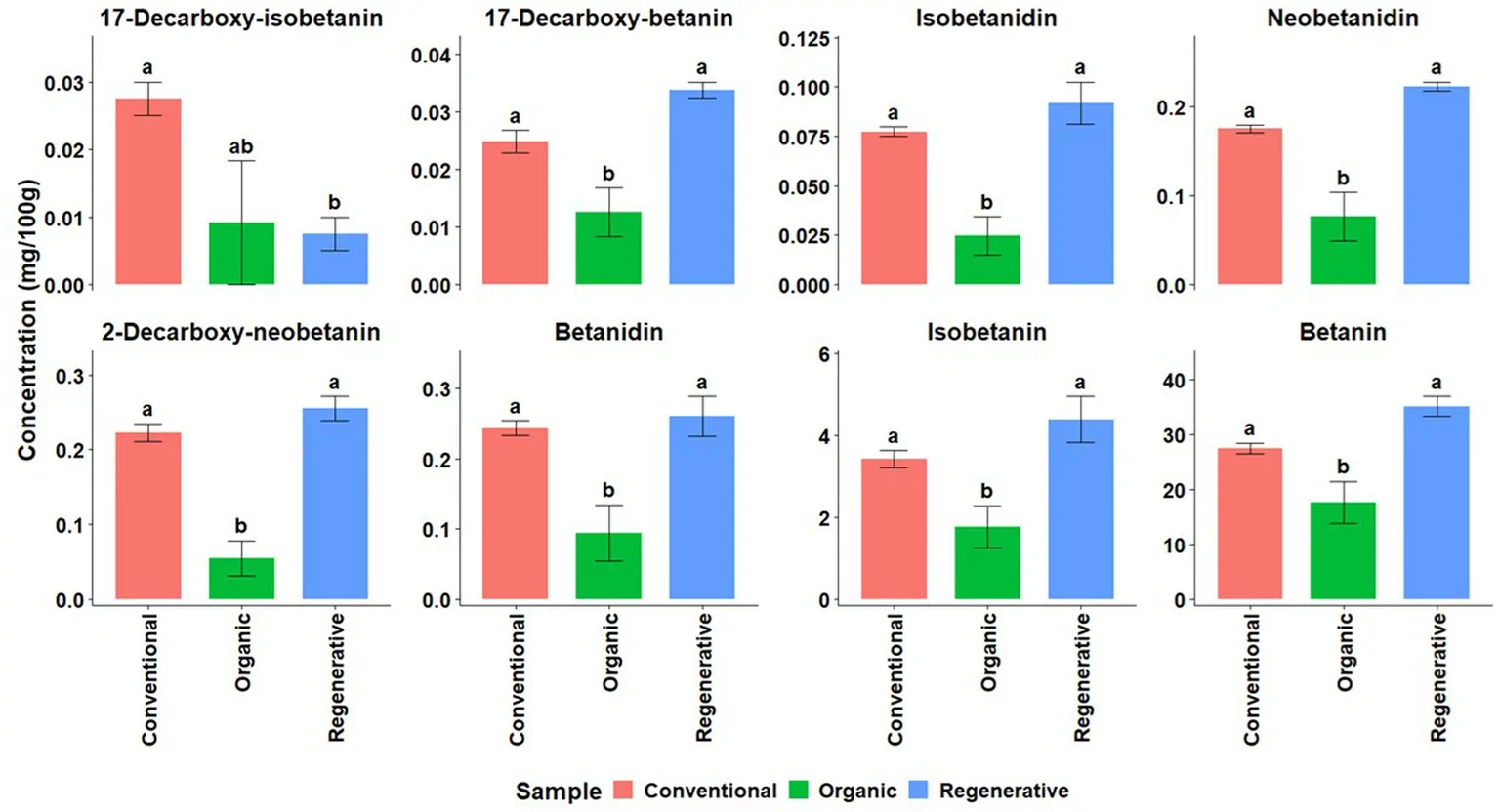
The bars indicate mean ± standard error of mean (*n* = 12) of individual betalain on fresh weight basis in conventional, organic, and ROC^™^ beetroots. Different lowercase letters indicate significant differences in betalain profile of beetroots based on Tukey–Kramer’s multiple comparison test (*α* ≤ 0.05).

In the present study, total betalains were highest in the ROC^™^ beetroots (40.43 ± 1.92 mg/100 g), although not significantly different from the conventional beetroots (31.75 ± 0.90 mg/100 g; *p* = 0.08), while the organic beetroots contained a significantly lower concentration of betalains (19.69 ± 4.29 mg/100 g) compared to both (*p* ≤ 0.01, Table 1). The concentrations of individual betalain compounds were significantly higher in conventional and ROC^™^ (all, *p* < 0.05) beetroots than in organic beetroots, except for 17-decarboxy-isobetanin, which was significantly lower in ROC^™^ beetroots than conventional (*p* < 0.05), while organic beetroots did not differ significantly from either conventional or ROC^™^ beetroots (*p* > 0.05; Figure 3). Higher accumulation of readily available nitrogen in conventional farming systems has been argued to be potentially responsible for producing abundant nitrogen-containing compounds such as betalains (24). This could partially explain the high levels of betalains in the conventional beetroots observed in this work, compared to the organic samples. The similarly high levels in the ROC^™^ could potentially be explained by the extensive cover cropping of nitrogen-fixing legumes in this system, which would also result in higher nitrogen availability for the subsequent beetroot crop. Our results are in contrast Carrillo et al. (5), who found higher betalains, alongside higher total antioxidant capacity, under organic production compared to conventional production. This discrepancy may be due to the differences in input and management practices, such as cultivar, maturity at harvest, environmental conditions or other site-specific factors, as both the genetic and growing-conditions-related factors have an influence on betalain accumulation (25).

**Table 1:** Composition of macro nutrients on fresh weight basis in beetroots grown under three different farming systems (*n* = 12 per group).

Nutrients	Conventional	Organic	ROC^TM^
Total protein (g/100 g)	2.1 ± 0.02^a^	2.1 ± 0.14^a^	1.8 ± 0.04^b^
Total nitrates (mg/100 g)	15.38 ± 0.27^c^	22.20 ± 1.38^a^	18.48 ± 0.45^b^
Total sugar (g/100 g)	11.31 ± 0.14^b^	14.15 ± 0.27^a^	14.19 ± 0.16^a^
Total (poly)phenols (μg/100 g)	3,260 ± 92.37^b^	4,981 ± 151.2^a^	4,974 ± 138.2^a^
Total betalains (mg/100 g)	31.75 ± 0.90^a^	19.69 ± 4.29^b^	40.43 ± 1.92^a^

Values are mean ± standard error of mean different superscript letters represent significant group differences; *p* < 0.05.

#### 3.1.2 Phenolics

Beetroots are also considered rich sources of diverse phenolic compounds, including simple phenols (such as ferulic, caffeic, and vanillic acids) and flavonoid polyphenols, which contribute to their color, flavor, and bioactivity (26). Phenolics exhibit potent antioxidant properties, helping to neutralize reactive oxygen species and reduce oxidative stress, a key factor in the development of chronic diseases (27, 28). In addition to their antioxidant activity, beetroot (poly)phenols have been associated with anti-inflammatory, cardioprotective, and potential anticancer effects through modulation of signaling pathways, inhibition of lipid peroxidation, and improvement of endothelial function (26). In the present study, total (poly)phenols were higher in organic (4,981 ± 151.2 μg/100 g) and ROC^™^ (4,974 ± 138.2 μg/100 g) beetroots compared to conventional beetroots (3,260 ± 92.37 μg/100 g) (all, *p* < 0.001; Table 1). ROC^™^ beetroots contained significantly higher levels of vanillic acid, catechol, and isoferulic acid than both conventional and organic beetroots (all, *p ≤* 0.002; Table 2). Additionally, ROC^™^ and organic beetroots exhibited significantly higher levels of the phenolic compounds vanillic acid 4-beta-D-glucopyranoside and 6-methylcoumarin, compared to conventional beetroots (all, *p* < 0.001; Table 2). Conversely, conventional beetroots exhibited significantly higher levels of the alkaloid trigonelline compared to the ROC^™^ and organic beetroots (all, *p* ≤ 0.004; Table 2).

**Table 2:** Phenolic concentration of top 20 phenolic compounds on fresh weight basis (*n* = 12) in conventional, organic, and ROC^™^ beetroots.

Metabolite (μg/g)	Conventional	Organic	ROC^TM^
3-hydroxybenzyl alcohol	0.00 ± 0.00^b^	0.00 ± 0.00^b^	10.15 ± 0.19^a^
L-kynurenine	4.06 ± 0.41^a^	2.43 ± 0.34^b^	1.16 ± 0.05^c^
Trigonelline	123.19 ± 5.34^a^	68.54 ± 6.22^c^	94.08 ± 6.23^b^
Syringic acid	24.07 ± 4.10^a^	16.91 ± 2.28^ab^	10.37 ± 0.70^b^
4-hydroxybenzyl alcohol	5.52 ± 0.44^a^	5.76 ± 0.40^a^	4.80 ± 0.21^a^
Vanillic acid	52.03 ± 3.98^b^	67.84 ± 4.97^b^	106.70 ± 8.45^a^
6-methylcoumarin	5.60 ± 0.23^b^	9.81 ± 0.60^a^	11.14 ± 1.02^a^
Isoferulic acid	0.00 ± 0.00^b^	0.00 ± 0.00^b^	2.31 ± 0.74^a^
Vanillic acid 4-β-D-glucopyranoside	689.90 ± 41.31^b^	1385.00 ± 97.31^a^	1346.00 ± 86.80^a^
m-coumaric acid	1.03 ± 0.03^b^	1.26 ± 0.05^a^	1.18 ± 0.02^a^
17-decarboxy-isobetanin	0.007 ± 0.003^a^	0.009 ± 0.009^ab^	0.028 ± 0.003^b^
Myrtillin	0.14 ± 0.05^a^	0.042 ± 0.009^b^	0.037 ± 0.008^b^
Indole-3-carboxaldehyde	2.89 ± 0.22^a^	3.42 ± 0.20^a^	2.96 ± 0.31^a^
Salicylic acid	2.83 ± 0.16^ab^	4.10 ± 0.61^a^	2.50 ± 0.12^b^
Catechol	0.00 ± 0.00^b^	0.00 ± 0.00^b^	1.25 ± 0.80^a^
Trans-ferulic acid	2209.00 ± 80.08^b^	3174.00 ± 85.45^a^	3216.00 ± 146.10^a^
Sinapic acid	0.59 ± 0.14^ab^	1.24 ± 0.53^a^	0.00 ± 0.00^b^
Rutin	0.14 ± 0.03^a^	0.11 ± 0.04^a^	0.12 ± 0.02^a^
Vitexin	2.11 ± 0.50^a^	2.73 ± 0.58^a^	2.40 ± 0.49^a^
3,4-dihydroxybenzoic acid	13.05 ± 1.03^b^	7.40 ± 1.83^b^	22.40 ± 2.43^a^

Values are presented as mean ± standard error of mean, different superscript letters represent significant group differences; *p* < 0.05.

Different lowercase letters indicate significant differences in antioxidant profile of beetroots based on Tukey–Kramer’s multiple comparison test (α ≤ 0.05).

Although, PLS-DA indicated clear separation in phenolic profiles of beetroots grown under different production systems (Figure 2a), the total polyphenol concentration was similar in both organic and ROC^™^ beetroots (*p* = 0.99) yet higher than conventionally grown beetroots (all, *p* < 0.001). Under conditions of high synthetic nitrogen availability, which can occur in conventional farming systems, plant tissues tend to show a decreased phenolic content as the plants allocate more resources towards protein synthesis and structural growth, and accumulate less total non-structured carbohydrates (TNC). In particular, TNCs are positively correlated with synthesis of phenolic compounds (29, 30). Additionally, high nitrogen inputs, which can occur in conventional systems, may decrease the synthesis of flavonoids and other phenolic compounds through the phenylpropanoid pathway (31).

#### 3.1.3 Nitrates

Total nitrate content was highest in the organic system (22.20 ± 1.38 mg/100 g), followed by ROC^™^ (18.48 ± 0.45 mg/100 g), while being lowest in conventional samples (15.38 ± 0.27 mg/100 g; Table 1). There is no consistent information available on the nitrate content in the beetroots from different production system, as some studies have reported higher nitrate contents in conventional (32) as well as organically produced beetroots (33). Additionally, they may vary considerably amongst beetroot samples, with values reported to range from 10.23 to 161.98 mg/100 g (34). Dietary nitrates are considered beneficial for human health as they can lower blood pressure and improve vascular function (35). While there is also a concern regarding high nitrate intakes, which have been linked to increased cancer risk (36), this is mainly the result of inorganic nitrate contamination in surface water and groundwater from agricultural and industrial runoff (37) as opposed to dietary intake from foods (such as beetroots). Median global intake of dietary nitrate is estimated to be 108 mg/day (38), and ranges from 58 to 218 mg/d, while human intervention studies generally suggest that dietary nitrate intakes in the range of 250–750 mg nitrate/day, can improve cardiovascular risk markers, particularly systolic blood pressure and endothelial function (39, 40). While the beetroots showed significant differences among the three farming systems, whether a ~ 7 mg difference between conventional and organic samples is meaningful depends on overall nitrate intake but absolute differences are modest and may not have immediate cardiovascular benefits (41).

#### 3.1.4 Minerals and macronutrients

The concentration of minerals in vegetables is affected by agronomic factors, including differences in soil nutrient profile, the interactions among nutrients in soil, fertilizer use, management practices, genetics of the vegetable, and weather conditions during the growing season (42–44). Various studies have shown either higher or similar mineral contents in vegetables grown in conventional systems compared to organic systems, likely because mineral fertilizer application is a common practice in conventional systems, making it less of a differentiator between production systems (45).

**Figure 4:**
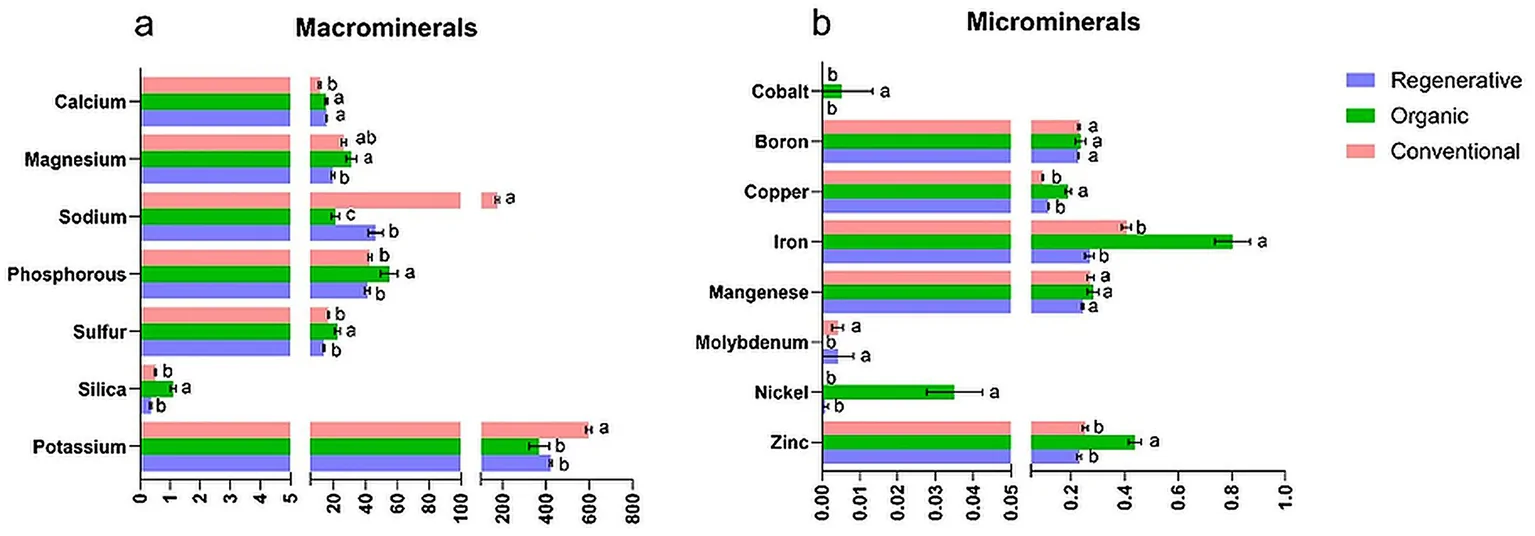
The bars indicate mean ± standard error of mean (*n* = 12) of macromineral **(a)**, micromineral **(b)** content on fresh weight basis in beetroots grown under conventional, organic, and ROC^™^. Different lowercase letters indicate significant differences in mineral profile of beetroots based on Tukey–Kramer’s multiple comparison test (*α* ≤ 0.05).

Among macro-minerals (Figure 4), we found conventional beetroots to contain significantly higher concentrations of potassium and sodium compared to ROC^™^ and organic beetroots (all, *p* ≤ 0.01). Further, phosphorus, silica, and sulfur were higher in organic compared to ROC^™^ and conventional beetroots (all, *p* ≤ 0.01). Although magnesium was not significantly different in conventional beetroots compared to ROC^™^ and organic beetroots (*p* = 0.08), magnesium was highest in organic beetroots and lowest in ROC^™^. Calcium concentrations were lower in conventional beetroots compared to the other systems (*p* < 0.001). Among microminerals, boron and manganese were present in similar concentrations in all three production systems, while organic beetroots had the highest concentrations of copper, iron, nickel, and zinc, while having the lowest concentration of molybdenum. Additionally, cobalt was only detected in organic beetroots. While we found differences in minerals between the three production systems, no single system was consistently superior across all minerals. The notable pattern was higher potassium in conventional beetroots, likely reflecting the NPK fertilizer application. Organic beetroots contained higher phosphorous, sulfur, silica, copper, nickel, and zinc. While ROC^™^ beetroots did not outperform other beetroot groups for any mineral.

Conventional beetroots contained lower sugar (11.31 ± 0.14 g/100 g) content compared to ROC^™^ (14.19 ± 0.16 g/100 g) and organic (14.15 ± 0.27 g/100 g; all, *p* < 0.001; Table 1). These results are in line with findings from Paulauskiene et al. (46), who reported higher sugar content in organic beetroots. The conditions within ROC^™^ and organic farming systems vs. conventional systems may have imposed conditions responsible for higher sugar content, along with other site-specific factors. Abiotic conditions can slow down growth and trigger carbohydrate translocation, causing increased production of sugars (47). Further, under conditions of limited nitrogen availability, excess carbon from photosynthesis can be rerouted towards the production of TNCs instead of protein synthesis (30). These mechanisms could partly explain the higher carbon retention in the form of sugars in organically and regeneratively produced beetroots compared to conventionally produced beetroots. Additionally, ROC^™^ beetroots had a significantly lower protein content (1.8 g/100 g) compared to conventional and organic (*p* < 0.001; Table 1). Previous studies indicate that conventionally grown crops tend to have higher protein contents (about 10–15% higher) as a result of nitrogen fertilizer application (48, 49). It must be noted that while differences exist between the beetroots from different farming systems, the absolute values at 1.8 and 2.1 g/100 g are modest to begin with.

### 3.2 Commercial beetroot powders

This work also studied commercial beetroot powders (*n* = 6) from five different brands comprising three production systems, including conventional (1 brand), organic (3 brands), and ROC^™^ (1 brand). These products reflect the real-world food system, as the raw material for these powders was grown on different farms with varying soils, climates, cultivars, and management practices. Therefore, the observed variation in nutrient composition could be driven by both differences in production systems as well as varying ecological conditions, and this is acknowledged as a limitation.

**Figure 5:**
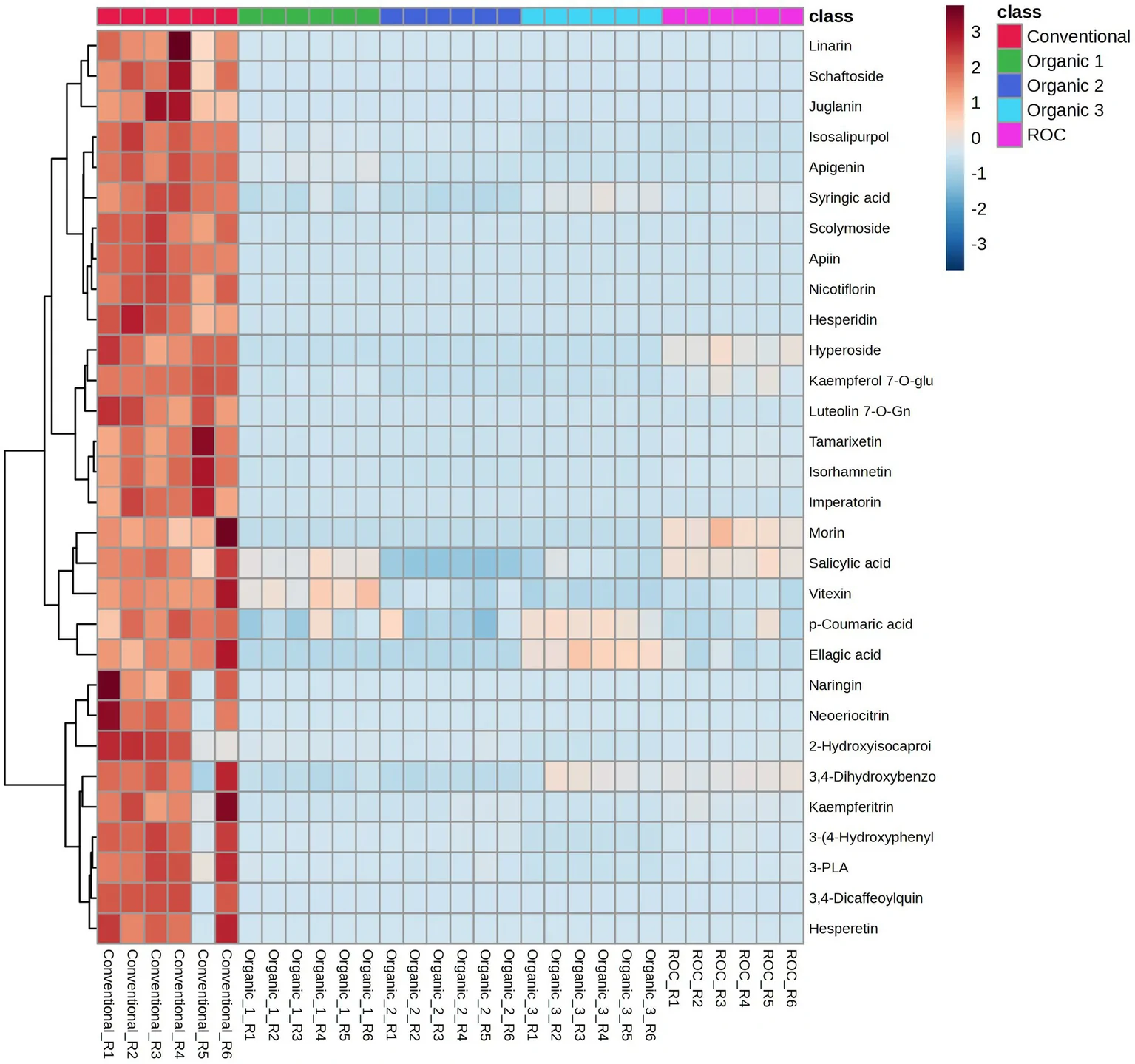
Heat maps illustrate the hierarchical clustering of top 30 plant secondary metabolites driving the group separation (polyphenols and betalains), among beetroot powders. The heat map was created using Euclidean distance as the distance metric, and Ward’s method as a clustering method based on partial least squares-discriminant analysis, variable importance in projection (PLS-DA VIP) score. Red (intensity ranges from 0 to 1.5) means higher abundance of the corresponding metabolite, whereas blue means lower abundance (intensity ranges from −1.5 to 0). The numbers below the heat map represent grouped samples (*n* = 6 for each group).

**Figure 6:**
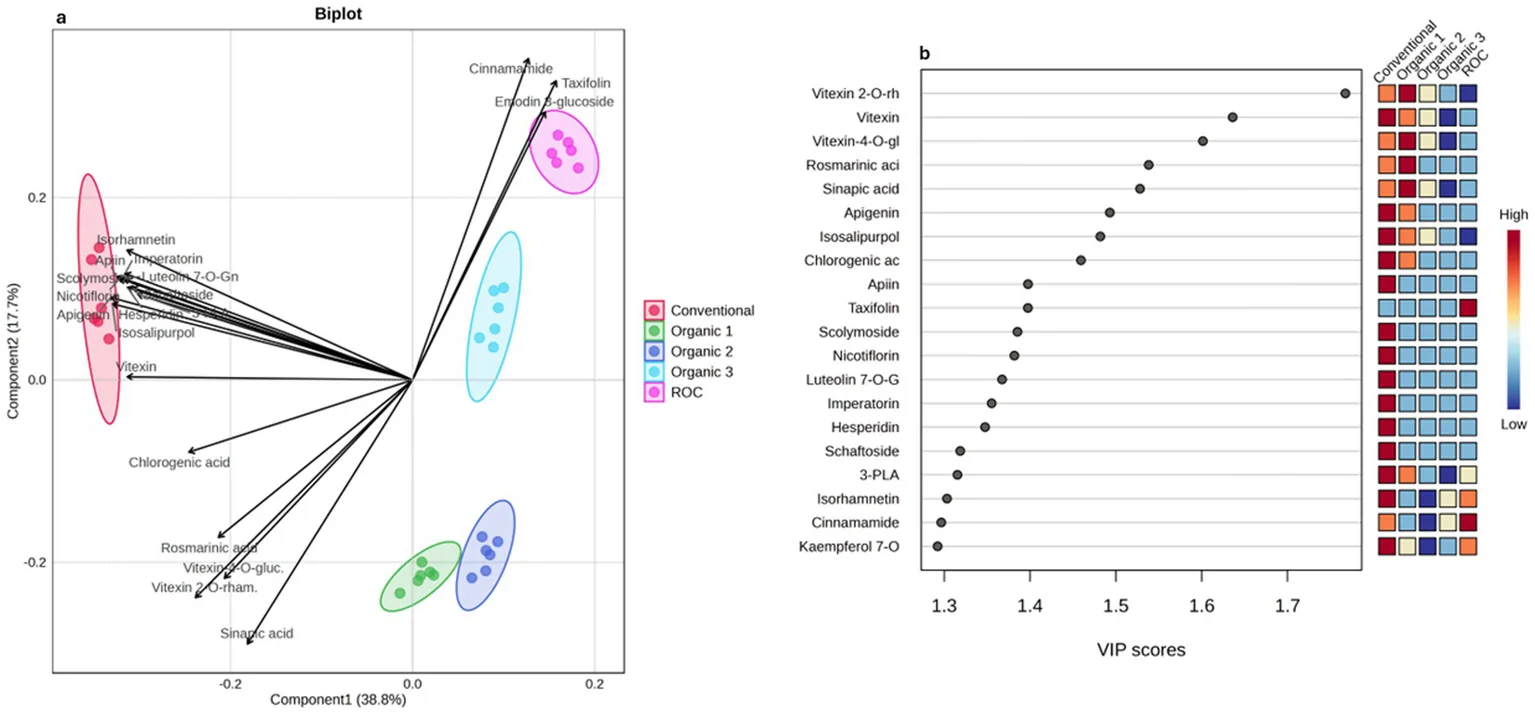
Partial Least Squares Discriminant Analysis (PLS-DA) biplot **(a)** based on the mean concentrations and **(b)** Variable Importance in Projection (VIP) score plot highlighting top 20 plant secondary metabolites driving the group separation, detected using UHPLC–MS/MS technique. The beetroot replications (colored dots) clustered together share similar metabolic profiles and species which are farther apart have distinct metabolic profiles.

Figure 5 presents the heatmap showing a total of 82 phenolics and related compounds, and eight betalains that were detected and quantified, highlighting the diverse profiles of plant secondary metabolites (polyphenols and betalains) among the beetroot powders from the conventional, organic, and ROC^™^ production systems. PLS-DA analysis of plant secondary nutrients indicated approximately 57% of total variance among beetroot powders, with component 1 explaining 38.8% variability and component 2 explaining 17.7% variability. This highlights the distinct phytonutrient profile among the powders (Figure 6a), indicating substantial variation amongst commercially available beetroot powder supplements. The PLS-DA biplot illustrates a stronger association of conventional powders with apigenin, apiin, isorhamnetin, scoparone, nicotiflorin, luteolin-7-O-glucoside, hesperidin, isosalipurpol, vitexin, and chlorogenic acid, while the ROC^™^ powder was distinctively rich in cinnamamide, taxifolin, and erysolin-glucoside. Organic powder 1 and 2 formed close clusters, indicating within-system variability but shared phenolic profiles, while organic powder 3 separated along component 2, reflecting the variabilities among organic powders despite the same production strategy. Even under the same umbrella of organic production, the variability in the plant secondary metabolite profile is not surprising, as beyond the production system, it is also determined by the uncontrollable or semi-controllable factors such as microclimate, soil type, and genotype (50). The VIP plot highlights the metabolites playing a key role in differentiating beetroot samples, and a higher VIP score indicates the metabolites have more significant roles in discriminating between different groups of beetroots (Figure 6b).

#### 3.2.1 Betalains

**Figure 7:**
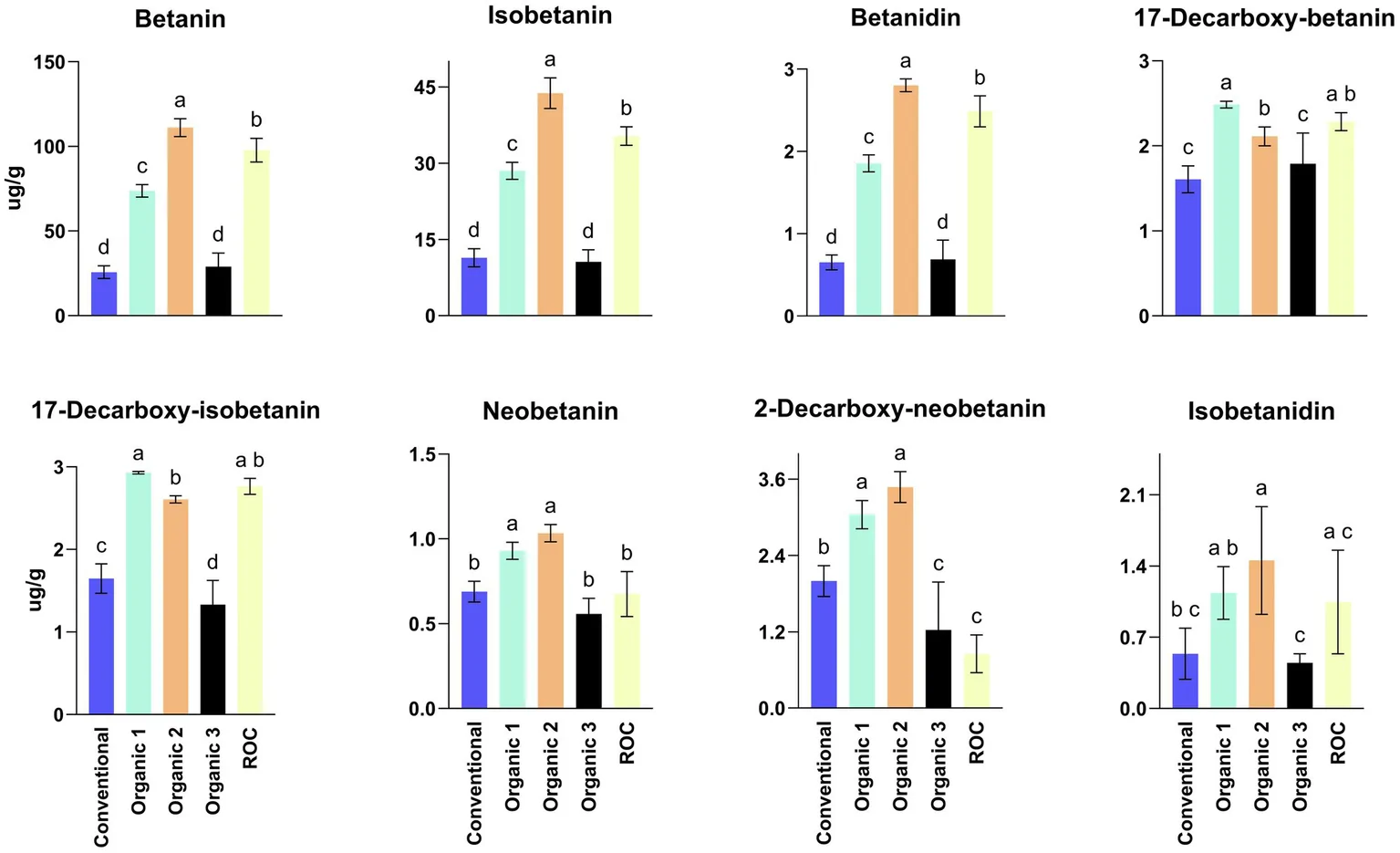
The bars indicate mean ± standard error of mean (*n* = 6) of in different beetroot powders. Different lowercase letters indicate significant differences in betalains profile of beetroots based on Tukey–Kramer’s multiple comparison test (*α* ≤ 0.05).

The individual betalain composition varied markedly among the beetroot powders. Total betalains were significantly higher in organic beetroot powder 2 compared to other powders, while organic beetroot powder 3 and conventional beetroot powder contained the lowest betalain levels (Table 3 and Figure 7). The wide range in betalain concentration among organic powders indicates the heterogeneity within the production system, which likely reflects brand-specific sourcing and processing differences. Organic beetroot powder 2 predominantly exhibited the highest concentrations of most of the betalains, although a few compounds displayed comparable or lower levels relative to other powders. Organic beetroot powder 1 exhibited the highest concentration of 17-decarboxy-betanin and 17-decarboxy-isobetanin and comparable concentrations of neobetanidin, 2-decarboxy-neobetanin, and isobetanidin to organic beetroot powder 2. The ROC^™^ beetroot powder contained high levels of 17-decarboxy-betanin and 17-decarboxy-isobetanin, which were on par with the values found in the organic beetroot powder 1. The remaining three products contained comparatively lower concentrations across most betalains (Figure 7).

**Table 3:** Composition of macro nutrients in beetroot powders grown under three different farming systems (*n* = 6 per group).

Nutrients	Conventional	Organic 1	Organic 2	Organic 3	ROC^TM^
Total protein (g/100 g)	13.77 ± 0.09^b^	13.30 ± 0.07^c^	7.78 ± 0.04^e^	10.02 ± 0.06^d^	14.99 ± 0.15^a^
Total nitrates (mg/100 g)	99.22 ± 1.34^b^	106.6 ± 2.00^b^	82.82 ± 5.61^c^	118.6 ± 1.40^a^	106.6 ± 1.3^b^
Total sugar (g/100 g)	69.07 ± 1.18^c^	84.47 ± 1.52^a^	84.47 ± 1.34^a^	79.17 ± 0.83^b^	83.53 ± 1.12^ab^
Total (poly)phenols (μg/100 g)	53,838 ± 5,097^a^	38,763 ± 1,385^b^	37,762 ± 117 ^b^	31,008 ± 1,094^b^	51,704 ± 1,334^a^
Total betalains (mg/100 g)	44.20 ± 2.45^d^	114.6 ± 2.23^c^	168.4 ± 3.33^a^	45.49 ± 4.77^d^	143.2 ± 3.45^b^

Values are presented as mean ± standard error of mean, different superscript letters represent significant group differences; *p* < 0.05.

#### 3.2.2 Polyphenols

Total (poly)phenols were significantly and similarly higher in the conventional (53,838 ± 5,097 μg/100 g) and ROC^™^ beetroot powder (51,704 ± 1,334 μg/100 g) compared to the organic powders (all, *p* ≤ 0.01), which contained similar concentration, varying between 31,008 ± 1,094 to 38,763 ± 1,385 μg/100 g (Table 3). Higher polyphenol concentrations in ROC^™^ crops are consistent with other data on regeneratively grown crops (7), however, ROC^™^ values still reflect the combined effect of practice, cultivar, and processing. Consistent with Wruss et al. (42), the variation among processed products is also reflective of the raw beetroot material and post-harvest handling across different brands, including cultivar differences, perhaps explaining why the conventional sample is higher than the organic sample and on par with the ROC^™^ sample.

The distinct profile of the top 30 plant secondary metabolites among the 82 compounds quantified in beetroot powders is displayed in Figure 5. Across the top 20 most-distinguishing (poly)phenols based on PLS-DA VIP score (Figure 6b), conventional beetroot powder showed the highest concentrations for compounds such as vitexin, kaempferol 7-O-glucoside, luteolin 7-O-glucoside, isosalipurpol, imperatorin, apigenin, isorhamnetin, schaftoside, 3-PLA, scolymoside, nicotiflorin, apiin, and hesperidin (all, *p* < 0.001, Table 4). Additionally, organic beetroot powder 1 contained amounts comparable to conventional beetroot powder for vitexin 4-O-glucoside, chlorogenic acid, sinapic acid, vitexin, 2-O-rhamnoside, and rosmarinic acid. The ROC^™^ beetroot powder contained the highest levels of cinnamamide and taxifolin among all beetroot powders (all, *p* < 0.001). Organic beetroot powder 3 also contained high concentrations of sinapic acid, similar to the conventional and organic powder 1 (Table 4). Although the biplot clearly distinguishes the conventional and ROC^™^ powders based on their polyphenolic and betalain composition, the total polyphenolic concentration was comparable between the two production systems. While organic management has been associated with higher concentrations of certain antioxidants in fresh produce (51), this study demonstrates that the production system alone does not guarantee uniformly higher phytonutrient content in processed products. These findings align with previous work indicating that nutrient availability, fertilization regime, and soil microbial activity—which differ markedly among conventional, organic, and regenerative systems—can influence carbon allocation between growth and secondary metabolism (52, 53).

**Table 4:** Phenolic concentration of top 20 phenolic compounds (*n* = 12) in conventional, organic, and ROC^™^ beetroot powders.

Metabolite (μg/g)	Conventional	Organic 1	Organic 2	Organic 3	ROC^TM^
17-decarboxy-betanin	1.61 ± 0.06^c^	2.49 ± 0.02^a^	2.11 ± 0.05^b^	1.79 ± 0.15^c^	2.29 ± 0.04^ab^
17-decarboxy-isobetanin	1.65 ± 0.07^c^	2.93 ± 0.01^a^	2.61 ± 0.02^b^	1.33 ± 0.12^d^	2.77 ± 0.04^ab^
2-decarboxy-neobetanin	2.00 ± 0.10^b^	3.04 ± 0.09^a^	3.48 ± 0.10^a^	1.23 ± 0.31^c^	0.85 ± 0.12^c^
3-PLA	352.60 ± 57.32^a^	24.20 ± 1.89^b^	4.74 ± 0.45^b^	19.81 ± 4.10^b^	20.94 ± 1.75^b^
Apigenin	49.97 ± 2.20^a^	4.77 ± 0.71^b^	0.00 ± 0.00^c^	0.00 ± 0.00^c^	0.00 ± 0.00^c^
Apiin	28.65 ± 1.32^a^	0.00 ± 0.00^b^	0.00 ± 0.00^b^	0.00 ± 0.00^b^	0.00 ± 0.00^b^
Betanidin	0.65 ± 0.04^d^	1.85 ± 0.04^c^	2.80 ± 0.03^a^	0.69 ± 0.10^d^	2.49 ± 0.08^b^
Betanin	25.67 ± 1.53^d^	73.68 ± 1.50^c^	111.10 ± 2.16^a^	28.88 ± 3.29^d^	97.78 ± 2.87^b^
Chlorogenic acid	21.94 ± 4.52^a^	15.64 ± 4.75^a^	0.00 ± 0.00^b^	0.00 ± 0.00^b^	0.00 ± 0.00^b^
Cinnamamide	111.60 ± 5.49^b^	42.49 ± 4.98^b^	68.87 ± 2.24^b^	12.97 ± 1.34^b^	822.40 ± 66.10^a^
Hesperidin	184.70 ± 22.15^a^	0.00 ± 0.00^b^	0.00 ± 0.00^b^	0.00 ± 0.00^b^	0.00 ± 0.00^b^
Imperatorin	117.60 ± 13.10^a^	0.00 ± 0.00^b^	0.00 ± 0.00^b^	0.00 ± 0.00^b^	0.00 ± 0.00^b^
Isobetanidin	0.54 ± 0.10^bc^	1.14 ± 0.11^ab^	1.46 ± 0.22^a^	0.45 ± 0.04^c^	1.05 ± 0.21^ac^
Isobetanin	11.40 ± 0.73^d^	28.49 ± 0.69^c^	43.79 ± 1.22^a^	10.56 ± 0.98^d^	35.36 ± 0.74^b^
Isorhamnetin	43.43 ± 4.34^a^	0.93 ± 0.10^b^	1.01 ± 0.09^b^	0.00 ± 0.00^b^	2.95 ± 0.31^b^
Isosalipurpol	26.34 ± 1.38^a^	2.00 ± 0.30^b^	0.49 ± 0.18^b^	1.49 ± 0.13^b^	0.10 ± 0.06^b^
Kaempferol 7-O-glucoside	145.00 ± 4.48^a^	9.76 ± 0.86^bc^	3.70 ± 0.45^c^	2.62 ± 0.35^c^	21.66 ± 4.54^b^
Luteolin 7-O-glucuronide	17.50 ± 1.69^a^	0.00 ± 0.00^b^	0.00 ± 0.00^b^	0.00 ± 0.00^b^	0.00 ± 0.00^b^
Neobetanin	0.69 ± 0.02^b^	0.93 ± 0.02^a^	1.03 ± 0.02^a^	0.56 ± 0.04^b^	0.68 ± 0.05^b^
Nicotiflorin	15.11 ± 1.16^a^	0.00 ± 0.00^b^	0.00 ± 0.00^b^	0.00 ± 0.00^b^	0.00 ± 0.00^b^
Rosmarinic acid	72.10 ± 14.42^a^	90.92 ± 3.44^a^	0.00 ± 0.00^b^	0.00 ± 0.00^b^	0.00 ± 0.00^b^
Schaftoside	10.28 ± 1.53^a^	0.00 ± 0.00^b^	0.00 ± 0.00^b^	0.00 ± 0.00^b^	0.00 ± 0.00^b^
Scolymoside	14.38 ± 1.02^a^	0.00 ± 0.00^b^	0.00 ± 0.00^b^	0.00 ± 0.00^b^	0.00 ± 0.00^b^
Sinapic acid	411.20 ± 17.56^a^	424.10 ± 29.39^a^	137.30 ± 4.83^b^	395.50 ± 36.06^a^	197.90 ± 13.42^b^
Taxifolin	0.00 ± 0.00^b^	0.00 ± 0.00^b^	0.00 ± 0.00^b^	0.00 ± 0.00^b^	8.11 ± 0.38^a^
Vitexin	245.80 ± 13.74^a^	303.30 ± 28.39^b^	55.91 ± 5.12^c^	112.10 ± 10.22^c^	71.14 ± 9.45^c^
Vitexin 2-O-rhamnoside	39.64 ± 2.15^a^	43.78 ± 3.58^a^	11.17 ± 0.70^bc^	17.19 ± 1.54^b^	5.50 ± 0.47^c^
Vitexin-4-O-glucoside	245.80 ± 13.74^a^	303.30 ± 28.39^b^	55.91 ± 5.12^c^	112.10 ± 10.22^c^	71.14 ± 9.45^c^

Values are presented as mean ± standard error of mean, different superscript letters represent significant group differences; *p* < 0.05.

Different lowercase letters indicate significant differences in antioxidant profile of beetroots based on Tukey–Kramer’s multiple comparison test (*α* ≤ 0.05).

#### 3.2.3 Nitrates

Among all beetroot powders evaluated, organic beetroot powder 3 contained the highest nitrate concentrations (118.60 ± 1.40 mg/100 g), exceeding all powders, while organic beetroot powder 2 contained the lowest (82.82 ± 5.61 mg/100 g). Conventional, ROC^™^, and organic beetroot powder 1 fell in similar ranges (99.22 ± 1.33 to 106.6 ± 2.00 mg/100 g), significantly different from both organic beetroot powders (Table 3). The variability is highly relevant to consumers as beetroot powders are commonly consumed for cardiovascular health and exercise performance benefit attributed to nitrate content (54). The results highlight that nitrate content in commercial powders is not standardized even within the same production system, and is likely the result of agroecological, cultivar., and post-harvest processing variations.

#### 3.2.4 Minerals and macronutrients

**Figure 8:**
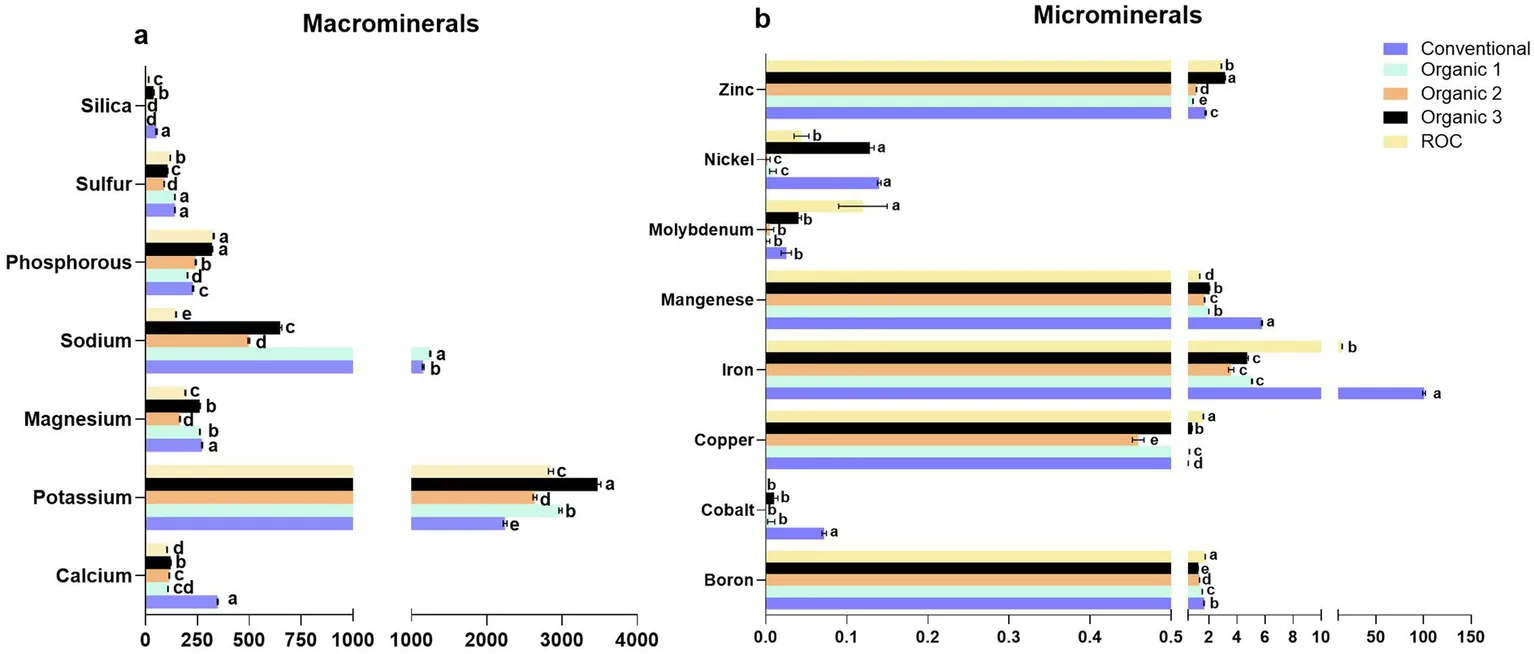
The bars indicate mean ± standard error of mean (*n* = 6) of macro-mineral **(a)** and micromineral **(b)** content in different beetroot powders. Different lowercase letters indicate significant differences in mineral profile of beetroots based on Tukey–Kramer’s multiple comparison test (*α* ≤ 0.05).

ROC^™^ and organic beetroot powder 3 contained the highest phosphorus concentrations among all products (*p* < 0.001). Organic 3 beetroot powder also had the highest potassium concentration (*p* < 0.001), while showing moderate levels of calcium, magnesium, and silica. The conventional beetroot powder exhibited the highest silica (*p* < 0.001), magnesium (*p* = 0.002), and calcium concentration (*p* < 0.001), while sulfur concentration was similar in both conventional and organic beetroot powder 1 (Figure 8a). Among microminerals, the conventional beetroot powder had significantly higher cobalt, iron, manganese, and nickel compared to other powders (all, *p* < 0.001). Organic beetroot powder 3 contained a comparable nickel concentration as the conventional powder (*p* = 0.54) while having the highest zinc concentration (*p* < 0.001). Organic beetroot powder 1 and 2 were on the low end across most measured micronutrients compared to other powders. The ROC^™^ beetroot powder contained the highest boron and copper (all, *p* < 0.001), with moderate iron and zinc (Figure 8b).

Organic beetroot powder 1 and 2 had the highest sugar (84.47 ± 1.52 and 84.47 ± g/100 g; respectively) content, exceeding organic 3 and conventional beetroot powder (all, *p* ≤ 0.001) and the conventional beetroot powder contained the least sugar (69.07 ± 1.18 g/100 g all, *p* < 0.001). ROC^™^ beetroot powder and organic beetroot powders 1 and 2 all fell in a similar range (~83–84 g/100 g; Table 3). The ROC^™^ beetroot powder showed the highest protein content (14.99 ± 0.15 g/100 g) followed by conventional beetroot powder (13.77 ± 0.09 g/100 g), organic beetroot powder 1 (13.30 ± 0.07 g/100 g), and organic beetroot powder 3 (10.02 ± 0.06 g/100 g), with organic beetroot powder 2 containing the lowest amount (7.78 ± 0.03 g/100 g; Table 3).

## 4 Limitations

Despite all fresh samples being sourced from the same growing season and from farms located in close proximity within Southwest Oregon, USA, to minimize potential confounding, several limitations should be acknowledged. The fresh beetroot samples were obtained from one local farmer per farming designation, with each farm intended to represent ROC^™^, organic, or conventional production under real-world conditions. This regional sourcing is a strength because it reduced geographic and seasonal variation among the fresh samples; however, this was not a controlled farming trial. As such, additional farm-level differences, including soil type, microclimate, water availability, cultivar selection, and other site-specific factors, may have contributed to the observed differences in beetroot composition. The commercial beetroot powders were also sourced from multiple brands, reflecting the heterogeneous supply chains that consumers encounter when purchasing processed beetroot products. These products are known to differ in geographic origin, climate, and soil condition and are likely to differ in cultivar selection and post-harvest processing. Therefore, without assessing the natural variability of each outcome across multiple farms within the same farming designation, the observed differences in fresh beetroots or beetroot powders cannot be attributed to farming practice alone. Nevertheless, this study provides insight into a real-world consumer scenario, in which fresh beetroots and commercial beetroot products are purchased from diverse production systems and supply chains. Larger studies that include multiple farms within each farming designation, across different seasons and geographic regions, are needed to more conclusively evaluate the relationship between farming practice and the biological composition of fresh beetroot and processed beetroot powders. Alternatively, a uniformly managed and well-controlled research site with ROC^™^, organic, and conventional systems represented within the same location could help isolate the effects of farming practice from other environmental and farm-specific sources of variation.

## 5 Conclusion

We found that fresh ROC^™^ and organic beetroots had higher phytonutrients, including phenolics, flavonoids, and nitrates compared to conventional beets, with no clear differences in primary nutrients such as minerals and protein. Our findings align with previous research reporting modest and sometimes inconsistent differences in mineral and protein concentrations between organic and conventional production systems (15) yet higher levels of phytonutrients (51). In particular, there is emerging evidence suggesting that the nutritional differences between regenerative, organic, and conventional crops are often more pronounced in phytonutrient profiles (e.g., phenolics, flavonoids, isoflavones) than in primary nutrient concentrations (6, 7, 55, 56).

This may be explained by the observation that primary nutrients (e.g., nitrogen and minerals) are often provided in both conventional and organic systems, albeit through synthetic fertilizers or organic amendments and cover crops, respectively—whereas polyphenols and other phytonutrients are not directly applied but are synthesized by plants in response to environmental interactions (57). In particular, studies have found higher polyphenol contents in vegetables when less synthetic nitrogen is added to the soil (56). Production systems with greater biodiversity, lower synthetic inputs, and/or stronger soil microbial activity, such as those typically found in organic and ROC^™^ systems, may therefore promote higher phytonutrient accumulation (6, 13).

Consistent with our results, other studies have observed higher levels of certain polyphenols in organic beetroots and other crops compared to conventionally grown counterparts. Commercial beetroot powders also showed substantial variability in phytochemical, nitrate, mineral, and macronutrient composition across conventional, organic, and ROC^™^ production systems. Multivariate analyses distinguished powders by production system, yet considerable within-system heterogeneity indicates that certification alone does not predict nutrient composition. Thus, the findings of our work, and those of others highlighted within this study, indicate a need for further nutritional profiling work in farms from different production systems. Future work should include a larger number of producers and crops as interest in regenerative agriculture continues to grow.

## Data Availability

The original contributions presented in the study are included in the article/Supplementary material, further inquiries can be directed to the corresponding author.

## Footnotes

1
https://www.metaboanalyst.ca/
